# Protein Food Matrix–ZnO Nanoparticle Interactions Affect Protein Conformation, but May not Be Biological Responses

**DOI:** 10.3390/ijms19123926

**Published:** 2018-12-07

**Authors:** Song-Hwa Bae, Jin Yu, Tae Geol Lee, Soo-Jin Choi

**Affiliations:** 1 Major of Food Science & Technology, Division of Applied Food System, Seoul Women’s University, Seoul 01797, Korea; songhwa29@naver.com (S.-H.B.); ky5031@swu.ac.kr (J.Y.); 2 Center for Nano-Bio Measurement, Korea Research Institute of Standards and Science (KRISS), Daejeon 34113, Korea; tglee@kriss.re.kr

**Keywords:** zinc oxide nanoparticles, interaction, protein, structural deformation, cytotoxicity, uptake, digestive efficiency

## Abstract

Because of their nutritional value, zinc oxide (ZnO) nanoparticles (NPs) are applied as a dietary source of zinc, by direct addition to complex, multiple-component food matrices. The thereby occurring interactions of NPs with food matrices may have biological or toxic effects. In particular, NP interactions with food protein can lead to structural deformation of the latter, potentially changing its digestive efficiency and gastrointestinal absorption. In this study, interactions between ZnO NPs and a representative complex protein food matrix, skim milk, were compared with those between NPs and individual components of this food matrix (i.e., protein, saccharide, and mineral). The effects of the interactions on biological responses were investigated in terms of cytotoxicity, cellular uptake, intestinal transport, structural deformation for proteins, and digestive efficiency. The results demonstrated that the physicochemical properties of ZnO NPs were strongly influenced by the protein matrix type, leading to an increased dispersion stability in the complex protein matrix. However, these interactions did not affect cell proliferation, membrane damage, cellular uptake, intestinal transportation, or protein digestive efficiency, although a slight conformational change of proteins was observed in the presence of ZnO NPs. In conclusion, no toxic effects were observed, suggesting the safety of NPs when added to complex food matrices.

## 1. Introduction

Zinc oxide (ZnO), a white insoluble powder, is used in diverse food products as a source of zinc because of its nutritional value [1,2,3,4]. Zinc is an essential trace element that plays a role in various cellular and immune functions [5,6,7,8]. ZnO has been listed as a food additive that is generally recognized as safe (GRAS) by the US Food and Drug Administration [9]. However, there is no specification for GRAS or food additive ZnO regarding particle size distribution. Advances in nanotechnology have led to the production of nanosized ZnO particles, which have a larger surface-area-to-volume ratio, possess a higher reactivity as well as different physicochemical properties. Many researches have demonstrated that ZnO nanoparticles (NPs) exhibited antimicrobial, anti-corrosive, and ultraviolet filtering properties as well as high nutrient absorption [5,10,11,12]. However, NPs may exert unexpected biological responses in comparison with the bulk-sized material [13,14,15]. The novel physicochemical characteristics of NPs have raised public concerns about their potentially undesirable effects on the human body.

When used as a dietary zinc supplement, ZnO NPs are added directly to food matrices that are composed of proteins, carbohydrates, lipids, minerals, and other trace nutrients. As a consequence, interactions between NPs and food components are very likely to occur, affecting the physicochemical properties of NPs and possibly inducing undesirable biological responses or even toxicity in humans. For example, enhanced gastrointestinal absorption of silica and titanium dioxide NPs in the presence of albumin or glucose has been reported [16,17]. Quantitative analysis of interactions between silica NP food additives and other food components demonstrated that saccharides, proteins, lipids, and minerals were adsorbed on the surface of silica NPs [18]. Moreover, these interactions were strongly affected by the presence of minor trace nutrients in the food matrices [18]. A recent report showed that ZnO NPs interacted differently with three different saccharide matrices: (1) acacia honey, constituting a complex matrix; (2) mixtures of equivalent amounts of fructose, glucose, sucrose, and maltose; and (3) monosaccharide (i.e., fructose or glucose) solutions; these interactions affected gastrointestinal absorption depending on the type of saccharide matrix [19]. These findings suggest that the interactions of NPs and food matrices are strongly influenced by the presence of other minor nutrients. In other words, NP interactions with complex food systems may differ from those with matrices consisting of only one component. Another important factor to be considered is that NP interactions with protein-containing foods can lead to structural deformation of the proteins [20,21,22], which may affect their digestive efficiency and gastrointestinal absorption [23]. However, few data are available about interactions between NP food additives and complex food systems. Moreover, the interaction effects on biological responses and conformation of food proteins have not been examined extensively.

Therefore, in the present study, we investigated interactions between ZnO NPs and skim milk, a complex protein-containing food matrix that also contains saccharides, minerals, and other trace elements; comparisons were made to ZnO NP interactions with matrices containing only one component (i.e., protein, saccharide, and mineral). The impact of the interaction effects on biological responses was examined for cell proliferation, cell membrane damage, cellular uptake, and intestinal absorption. Finally, possible changes in structural deformation and digestive efficiency of proteins in the presence of ZnO NPs were also evaluated.

## 2. Results

### 2.1. Characterization of ZnO NPs in Food Matrices

Scanning electron microscopy (SEM) showed that ZnO NPs generally had spherical shapes with a primary particle size of 25.4 ± 9.3 nm [19]. However, the hydrodynamic radii of NPs in an aqueous solution (distilled water, DW), measured by dynamic light scattering (DLS), were 1957.0 ± 113.3 nm, revealing agglomerate or aggregate states (Table 1). On the other hand, the hydrodynamic diameters of ZnO NPs dispersed in skim milk (1123.0 ± 266.1 nm) or lactose solution (1509.0 ± 462.1 nm) were significantly decreased relative to NPs prepared in casein (2417.0 ± 744.1 nm) or calcium solutions (3186.4 ± 319.6 nm) after dilution in DW for DLS analysis. A similar tendency was observed when the hydrodynamic radii of NPs dispersed in food matrices were measured after dilution in Minimum Essential Medium (MEM), representing typical cell culture conditions. The strongest decreases in hydrodynamic radius of ZnO NPs were found after their dispersion in MEM.

The positive value of the zeta potential of ZnO NPs in DW (14.8 ± 0.7 mV) changed to negative after dispersion in skim milk or casein solutions, whereas it remained positive in lactose or calcium solutions (Table 1). It is worth noting that these zeta potentials were observed after dilution in DW. On the other hand, zeta potential values of ZnO NPs dispersed in food matrices, followed by dilution in MEM, were found to be negative in all cases, ranging from ~−9 to −10 mV.

On the other hand, solubility of ZnO NPs in DW, MEM, skim milk, casein, lactose, or calcium solutions was less than 1%, without statistical difference (*p* > 0.05).

### 2.2. Interactions between ZnO NPs and Protein Matrices

**Figure 1:**
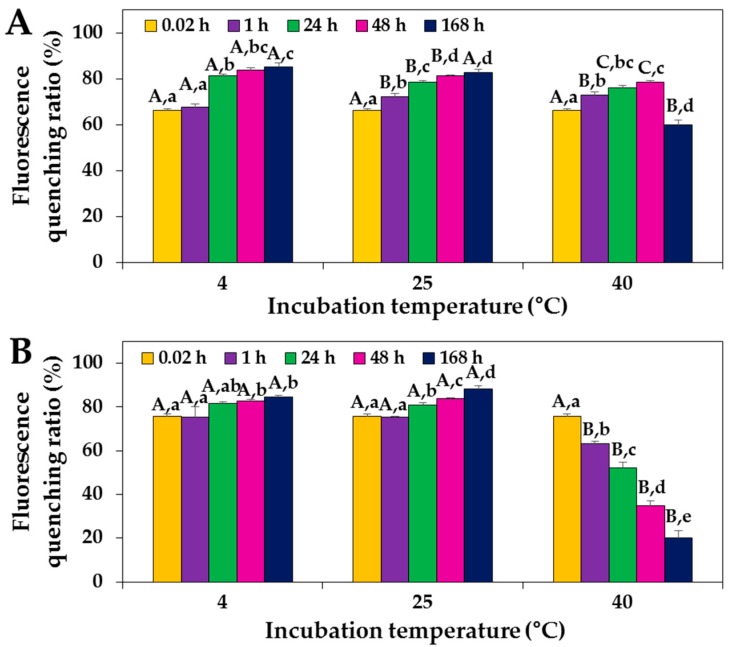
Fluorescence quenching ratios of (**A**) skim milk and (**B**) casein solutions incubated with ZnO NPs at 4, 25, and 40 °C. Capital letters (A–C) indicate significant differences in quenching for different incubation temperatures (*p* < 0.05). The lower-case letters (a–e) indicate significant differences between incubation times (*p* < 0.05).

Fluorescence quenching analysis was performed to characterize ZnO NP interactions with proteins, since proteins contain tryptophan residues that exhibit maximum fluorescence at 340 nm. The solution of casein, the major protein component of skim milk, was adjusted to have an equivalent concentration (~35%) as that of skim milk. The results show that high fluorescence quenching ratios (i.e., 66–76% quenching) of skim milk and the casein solution were observed immediately after adding ZnO NPs to the protein solutions (Figure 1). The fluorescence quenching ratios significantly increased as time increased for both skim milk and casein solutions after incubation with ZnO NPs at temperatures of 4 and 25 °C. However, at 40 °C, a significant weakening of fluorescence quenching in skim milk was induced by ZnO NPs after 168 h, and a time-dependent decrease was seen after ZnO NP incubation of the casein solution over 168 h.

Interestingly, moderately high quenching ratios were found in casein solution in comparison with those in skim milk at 4 and 25 °C; however, no different interaction effect was observed at the different incubation temperatures.

### 2.3. Interactions between ZnO NPs and Saccharide in Protein Matrix

**Figure 2:**
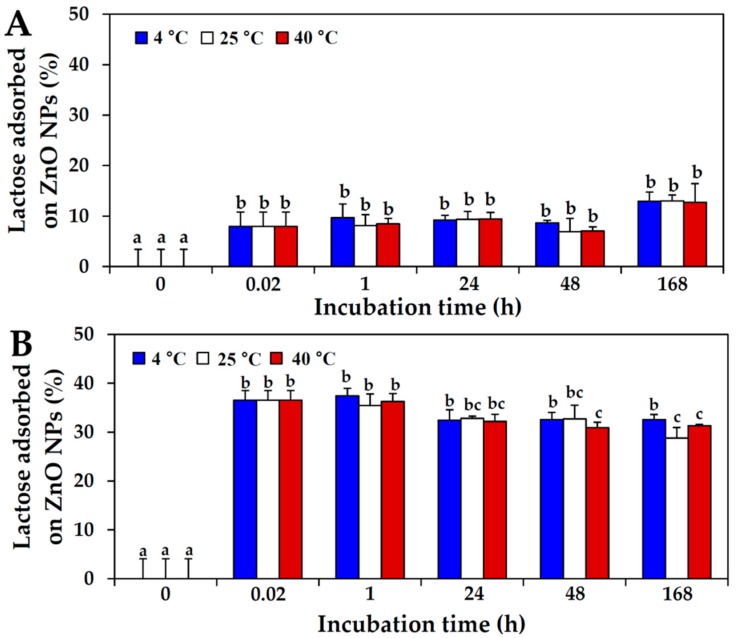
High-performance liquid chromatography (HPLC) analysis of adsorbed lactose in (**A**) skim milk and (**B**) lactose solutions on ZnO NPs after incubation times of up to 168 h at 4, 25, and 40 °C. Different lower-case letters (a–c) indicate significant differences between incubation times (*p* < 0.05). No significant differences between incubation temperatures were found (*p* > 0.05).

Since the complex protein matrix, skim milk, also contains a saccharide, lactose (~50%) as a major component, ZnO NP interactions with lactose in skim milk or lactose-only solution were evaluated by quantifying the amount of lactose adsorbed onto the surface of NPs using high-performance lipid chromatography (HPLC). The lactose concentration was adjusted to 50%, i.e., a level equivalent to that in skim milk. Figure 2A shows that about ~8–13% of the lactose in skim milk interacted with ZnO NPs, whereas ~29–37% of lactose in the lactose-only solution did so (Figure 2B). The effect of incubation time on these interactions was minor, and no significant effects were observed of different incubation temperatures (*p* > 0.05).

### 2.4. Interactions between ZnO NPs and Mineral in Protein Matrix

**Figure 3:**
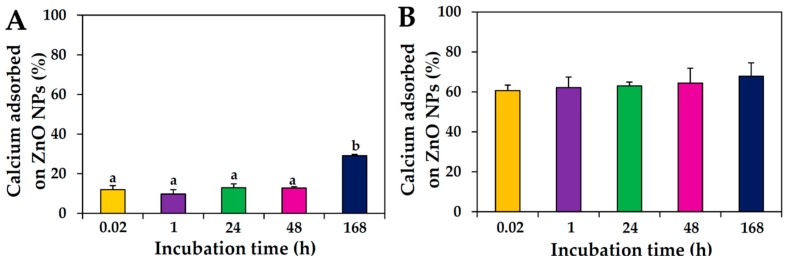
Inductively coupled plasma-atomic emission spectroscopy (ICP-AES) analysis of calcium adsorption to ZnO NPs after incubation times up to 168 h at 25 °C in (**A**) skim milk and (**B**) calcium-only solutions. Different lower-case letters (a, b) in Figure 3A indicate significant differences after different incubation times (*p* < 0.05). No significant differences between incubation times were found (*p* > 0.05).

NP interactions with the most abundant mineral in skim milk, calcium (~1.4%), were also evaluated and compared with those in calcium-only solution. The results showed that between 10% and 29% of the calcium in skim milk was adsorbed on the surface of ZnO NPs upon incubation, whereas more than 60% of the calcium in calcium-only solution was found to interact with NPs immediately after addition of the NPs; no significant effects were found of different incubation (*p* > 0.05; Figure 3).

### 2.5. Primary Structural Stability of Proteins

**Figure 4:**
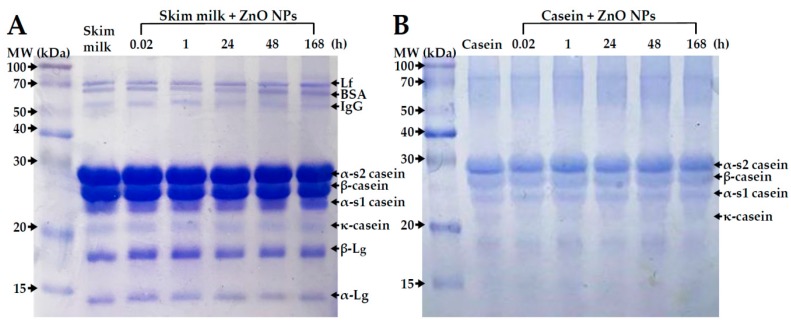
Sodium dodecyl sulfate polyacrylamide gel electrophoresis (SDS-PAGE) of (**A**) skim milk and (**B**) casein solutions in the absence or presence of ZnO NPs for 168 h at 25 °C. MW, molecular weight; Lf, lactoferrin; BSA, bovine serum albumin; IgG, immunoglobulin G; Lg, lactoglobulin.

The primary structure of proteins in the absence or presence of ZnO NPs was evaluated by sodium dodecyl sulfate polyacrylamide gel electrophoresis (SDS-PAGE), based on molecular weights of proteins depending on amino acid compositions. No remarkable primary structural change was observed in both skim milk and casein incubated with ZnO NPs even after 168 h (Figure 4). 

### 2.6. Conformational Stability of Proteins

**Figure 5:**
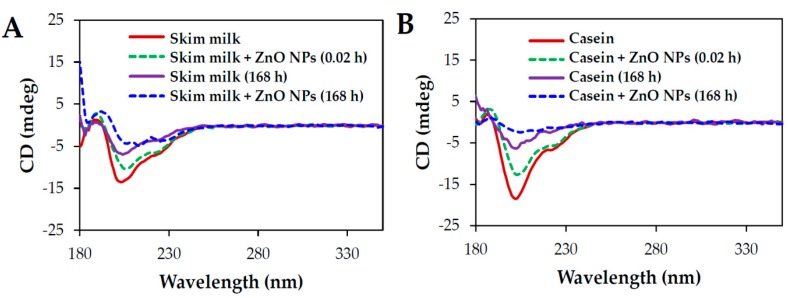
Circular dichroism (CD) analysis of (**A**) skim milk and (**B**) casein solutions in the absence or presence of ZnO NPs for 168 h at 25 °C.

The stability of the secondary structure of protein in skim milk or casein solution upon NP interaction was investigated by circular dichroism (CD) spectroscopy. The results showed that both skim milk and casein solution displayed typical α-helical secondary structures, producing two negative characteristic bands at 208 and 222 nm (Figure 5). The ellipticity of skim milk and casein solution decreased with time for up to 168 h after incubation at room temperature.

When the skim milk and casein solutions were incubated with ZnO NPs, decreases in ellipticity were observed only immediately after incubation, although the two α-helical bands at 208 and 222 nm could still be observed in both solutions. On the other hand, especially for casein solution, α-helical bands were no longer evident, virtually disappearing after incubation with NP incubation for 168 h.

### 2.7. Cytotoxicity

**Figure 6:**
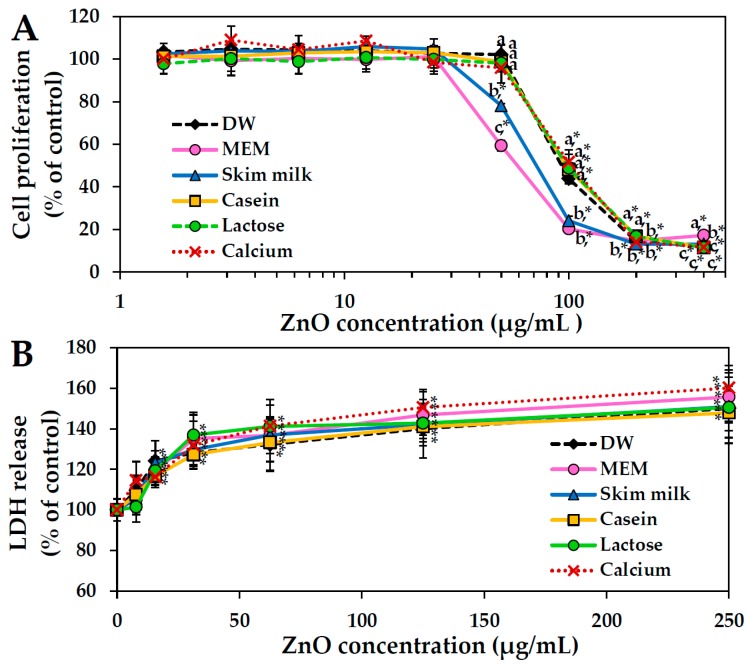
Effects of ZnO NPs suspended in distilled water (DW), Minimum Essential Medium (MEM), skim milk, and solutions of casein, lactose, or calcium on (**A**) cell proliferation and (**B**) membrane damage of human intestinal Caco-2 cells after a 24 h incubation. Different lower case letters (a–c) in Figure 6A indicate significant differences between dispersed matrices (*p* < 0.05). * Significant differences with untreated controls (*p* < 0.05). No statistical differences were found between dispersed matrices (*p* > 0.05).

The effects of ZnO NP interactions with food matrices on cell proliferation and membrane damage were investigated in human intestinal epithelial Caco-2 cells. The results showed that dispersion of ZnO NPs in cell culture medium (MEM) produced the strongest inhibition of cell proliferation, whereas NPs in skim milk had a similar but more modest effect (Figure 6A). No significant differences were seen in dispersions of ZnO NPs in casein, lactose, calcium, and DW (*p* > 0.05).

Furthermore, no effects were observed of interactions between ZnO NPs and food matrices on membrane damage, as measured by lactate dehydrogenase (LDH) leakage assay (Figure 6B).

### 2.8. Cellular Uptake

**Figure 7:**
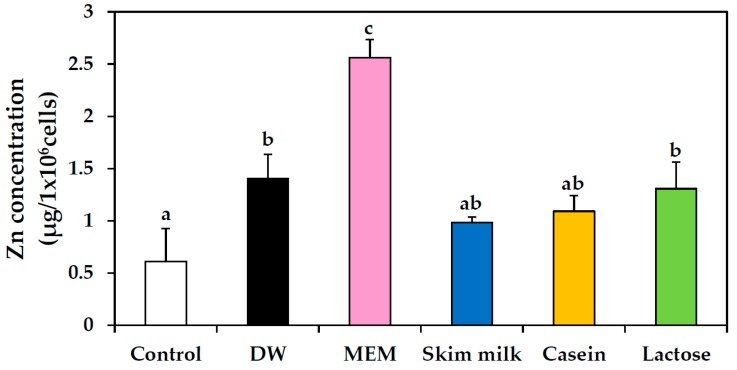
Cellular uptake of ZnO NPs dispersed in distilled water (DW), Minimum Essential Medium (MEM), skim milk, casein, or lactose solutions in human intestinal Caco-2 cells after a 6 h incubation, as determined by inductively coupled plasma-atomic emission spectroscopy (ICP-AES). Significant differences between results for the various dispersed matrices are indicated by different lower-case letters (a–d; *p* < 0.05).

Intracellular internalization of ZnO NPs prepared in food matrices was measured and analyzed using inductively coupled plasma-atomic emission spectroscopy (ICP-AES) analysis, as shown in Figure 7. No significant increases in cellular uptake were found between ZnO NPs suspended in DW, skim milk, casein, and lactose solutions; in contrast, significant increase was observed in internalized NPs suspended in MEM. Cellular uptake of ZnO NPs suspended in calcium solution was not evaluated in detail, because MEM already contains calcium.

### 2.9. Intestinal Transport

**Figure 8:**
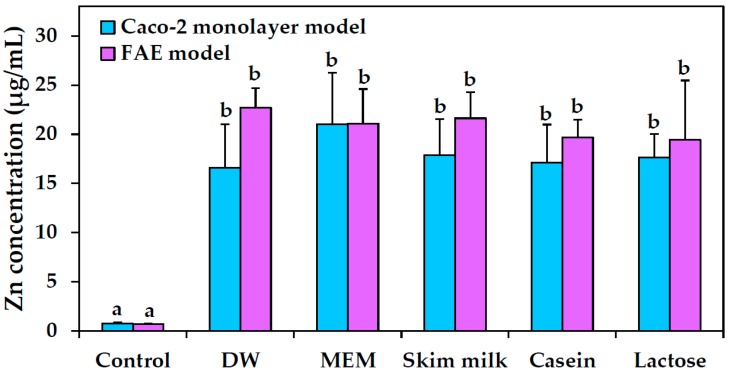
Intestinal transport of ZnO NPs using in vitro models of Caco-2 monolayers and human follicle-associated epithelium (FAE) after a 6 h treatment. Significant differences between dispersed matrices are indicated by different lower-case letters (a, b) (*p* < 0.05). No statistical differences between results from Caco-2 monolayer and FAE models were found (*p* > 0.05).

Intestinal transport amounts of ZnO NPs in various food matrices were measured with ICP-AES analysis, as shown in Figure 8. Two different in vitro models were used—human intestinal follicle-associated epithelium (FAE) and Caco-2 monolayers—representing intestinal microfold (M) cells in Peyer’s patches and tight junctions in the small intestine, respectively. The results showed no significant increases in intestinal transport between ZnO NPs suspended in DW, MEM, skim milk, casein, and lactose solutions.

### 2.10. Digestive Efficiency of Proteins

**Figure 9:**
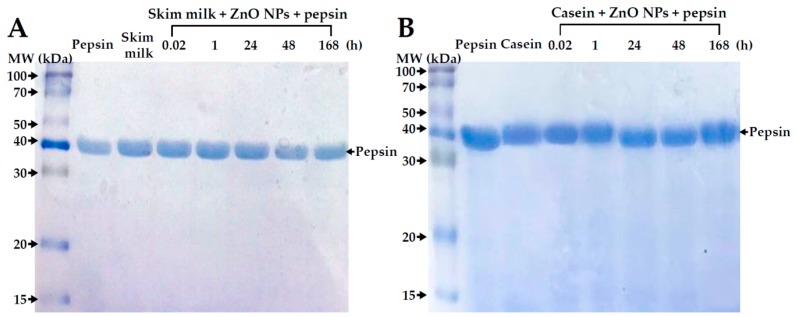
Sodium dodecyl sulfate polyacrylamide gel electrophoresis (SDS-PAGE) of (**A**) skim milk and (**B**) casein in the absence or presence of ZnO NPs after incubation times of up to 168 h at 25 °C, followed by a 1 h treatment with pepsin (1990 units/mL).

The digestion of skim milk or casein solutions treated with ZnO NPs was evaluated under simulated gastric conditions and analyzed by SDS-PAGE. Figure 9 shows that skim milk and casein solutions was normally degraded in gastric fluid after a 168 h interaction with ZnO NPs; no protein bands were observed, except for the gastric digestive enzyme, pepsin.

## 3. Discussion

The present study investigated interactions between ZnO NPs and a complex protein-containing food matrix, skim milk, composed of ~35% protein, ~50% lactose, lipids, minerals, and individual amino acids, to elucidate effects of NP food additives that are added directly to complex, multi-component food systems. ZnO NP interactions with the major protein, saccharide, and mineral components in skim milk, i.e., casein, lactose, and calcium, were also evaluated to clarify whether NPs interact differently with complex food matrices compared with matrices containing only one component.

The physicochemical characterization of ZnO NPs revealed that the positive zeta potentials of NPs in DW shifted to negative values in skim milk or casein solution, but not in lactose or calcium solutions (Table 1), which suggests that negatively charged casein (isoelectric point at pH ~4.6) under neutral conditions determines the shift in zeta potential change of the NPs. A positive zeta potential of ZnO NPs was maintained in lactose solution, which is in line with their positive charge observed in fructose or glucose solutions [19]. On the other hand, zeta potentials of ZnO NPs prepared in protein matrices, lactose, or calcium solution were all negative (between −9 and −10 mV) after dilution in MEM for zeta potential measurement. This observation reflects the zeta potential change under cell culture conditions, implying that the surface charge of ZnO NPs is not a critical factor affecting cellular responses.

The DLS data showed that ZnO NPs were agglomerated or aggregated in DW and that protein matrices, lactose, or calcium differently affected the hydrodynamic radii of ZnO NPs (Table 1). In particular, the hydrodynamic radii were decreased considerably in skim milk, but not in casein solution, indicating that minor nutrients and other components in skim milk affect NP–food matrix formation. Since ZnO NPs tend to form aggregates in DW, NP interactions with complex protein matrices, rather than with only casein, may contribute significantly to particle dispersion. This is presumably via electrostatic repulsion due to the presence of minor minerals and individual amino acids, which can prevent agglomeration or aggregation by separating NPs from each other and from surfaces [24,25,26,27,28,29]. On the other hand, many reports have demonstrated the agglomerate or aggregate fate of ZnO NPs in aqueous solution [19,24,25,26]. Reduced hydrodynamic radii of ZnO NPs in lactose solution also suggest that saccharides may act as dispersants [19,30,31,32,33]. Meanwhile, the hydrodynamic radii of ZnO NPs dispersed in food matrices showed a similar tendency when diluted in MEM for DLS analysis, while showing reduced diameters in skim milk or lactose solution. It is worth noting that remarkably small hydrodynamic radii of ZnO NPs in MEM were observed, in line with the role of cell culture medium or fetal bovine serum (FBS) as dispersants [34,35,36,37]. The results indicate that the interactions between ZnO NPs and food matrices affect hydrodynamic radii of NPs, but not their zeta potential values under cell culture conditions. On the other hand, ZnO NP interactions with food matrices did not affect solubility.

ZnO NP–protein interactions were clearly demonstrated by high fluorescence quenching ratios, just after adding NPs to protein solutions (Figure 1). Slightly high fluorescence quenching at 4 and 25 °C and remarkably low quenching at 40 °C were observed in NPs in casein solution relative to NPs in skim milk, implying different ZnO NP interactions depending on the type of protein matrix. Although low fluorescence quenching ratios at 40 °C were probably associated with protein denaturation, the complex protein matrix, skim milk, seems to be more resistant against protein denaturation. It should be noted that the casein concentration in the solution was adjusted to have the same amount as in skim milk.

ZnO NP interactions with lactose were also found to be strongly affected by the presence of other nutrients in skim milk, as observed by the 8–13% interaction with lactose in skim milk compared with the 29–37% interaction in the lactose-only solution (Figure 2). In the case of ZnO NP interactions with the most abundant mineral in skim milk, calcium, high NP interactions with calcium-only solution (~60%) were found compared with those with calcium in skim milk (10–29%) (Figure 3). Taken together, these results suggest that ZnO NPs interacted more strongly with a matrix composed of only one component, whereas the more complex protein matrix, skim milk, contributed to NP dispersion, as shown by the considerably reduced hydrodynamic radii of ZnO NPs (Table 1). Put otherwise, the interactions between ZnO NPs and food components may be weaker in complex food systems. Hence, the likely driving force guiding interactions between ZnO NP and components in skim milk is electrostatic attraction rather than strong adsorption on NP surface, ultimately contributing to dispersion stability.

ZnO NP interactions with casein or skim milk was found to induce a slight deformation of proteins’ secondary structure, as evidenced by CD spectroscopy (Figure 5), but not by data on primary structural stability (Figure 4). The two α-helical bands at 208 and 222 nm were maintained just after adding ZnO NPs to the protein solutions. A clear structural deformation was observed after NP incubation for 168 h, even in comparison with skim milk or casein controls without ZnO NPs. Structural deformation was stronger in the casein solution than in skim milk treated with ZnO NPs for 168 h, suggesting an additional role of other minor components in skim milk against protein denaturation, as confirmed by the fluorescence quenching results (Figure 1).

The NP interactions were associated with a slight inhibition of cell proliferation when ZnO NPs were dispersed in skim milk, but not in casein, lactose or calcium solutions (Figure 6A). Cell proliferation was inhibited more strongly by ZnO NPs in MEM, in which hydrodynamic radii of NPs were found to be reduced considerably as shown by DLS results (Table 1). Hence, this result is likely related to dispersion stability. However, ZnO NP interactions with the different food matrices did not affect membrane damage (Figure 6B) or cellular uptake (Figure 7) of the NPs. The cellular uptake of ZnO NPs was found to be most prominent after dispersion in MEM, which is known to support high dispersion stability as shown by DLS results (Table 1). Furthermore, intestinal transport and mechanism of ZnO NPs were not affected differently by the different food matrices (Figure 8). It is likely that ZnO NPs interacted with food matrices, but these interactions were too weak to affect cytotoxicity, cellular uptake, and intestinal transport rates. It is noteworthy that that digestive efficiency of both skim milk and casein was not affected by the interactions under simulated gastric conditions (Figure 9), in spite of slight deformations in secondary (Figure 5), but not primary (Figure 4) structures of proteins, induced by ZnO NP. It is likely that structural deformations caused by ZnO NPs were weak, and probably reversible, so that the digestive efficiency of skim milk and casein solution was not affected.

## 4. Materials and Methods

### 4.1. Materials

ZnO NPs were purchased from Sumitomo (Tokyo, Japan) and prepared in DW (stock suspension, 5 mg/mL) for 30 min prior to experiments. Materials used were as follows: casein sodium salt from bovine milk (Sigma-Aldrich, St. Louis, MO, USA), skim milk (Seoul Milk, Seoul, Korea), D-(+)-lactose (Sigma-Aldrich), CaOH_2_ (Yakuri Pure Chemicals Co., Ltd., Osaka, Japan), calcium standard solution (Sigma-Aldrich), and pepsin from porcine gastric mucosa (622 units/mg; Sigma-Aldrich).

### 4.2. Characterization of ZnO NPs in Food Matrices

Stock suspension of ZnO NPs (5 mg/mL) was dispersed in skim milk (1 mg/mL), casein (0.35 mg/mL), lactose (0.5 mg/mL), or calcium (14 μg/mL as calcium in Ca(OH)_2_) solutions. Casein and lactose concentrations were adjusted to equivalence based on the contents from skim milk. Primary particle size and morphology were determined by SEM (FEI Quanta 250 FEG, Hillsboro, OR, USA). Zeta potentials and hydrodynamic diameters of ZnO NPs prepared in DW or food matrices were measured with a Zetasizer Nano System (Malvern Instruments, Worcestershire, UK).

### 4.3. Solubility of ZnO NPs in Food Matrices

ZnO NPs (5 mg/mL) were dispersed in DW, MEM, skim milk (1 mg/mL), casein (0.35 mg/mL), lactose (0.5 mg/mL), or calcium (14 μg/mL as calcium in Ca(OH)_2_) solutions, respectively. After incubation times of up to 6 h at 25 °C, the supernatants were collected by ultracentrifugation (16,000× *g*) for 15 min, filtered through a syringe filter (0.45 μm; Advantech, Taipei, Taiwan), and digested as described for the ICP-AES analysis.

### 4.4. Interactions between ZnO NPs and Protein Matrices

The stock suspension of ZnO NPs was suspended in skim milk (1 mg/mL) or casein solution (0.35 mg/mL), obtaining a final NP concentration of 0.5 mg/mL, and incubated with gentle shaking at 4, 25, and 40 °C. After incubation for 0.02, 1, 24, 48, and 168 h, protein fluorescence quenching analysis was carried out using a luminescence spectrometer (SpectraMax^®^ M3; Molecular Devices, Sunnyvale, CA, USA). The excitation wavelength was 280 nm, and fluorescence emission was scanned at wavelengths between 300 and 420 nm. Fluorescence quenching ratios were calculated by the following equation: (I_0_ − I)/I_0_, with I_0_ and I representing basal fluorescence emission intensity without and with NPs, respectively.

### 4.5. Interactions between ZnO NPs and Saccharide in Protein Matrix

The stock suspension of ZnO NPs was suspended in skim milk (1 mg/mL) or lactose solution (0.5 mg/mL), obtaining a final NP concentration of 0.5 mg/mL, and incubated with gentle shaking at 4, 25, and 40 °C. After incubation for 0.02, 1, 24, 48, and 168 h, protein precipitation was performed using Carrez Clarification Reagent Kit (Sigma-Aldrich, St. Louis, MI, USA) according to the manufacturer’s protocol. After centrifugation at 10,000× *g*, the supernatants were collected and filtered by syringe filter (0.22 μm; Agela Techonologies Inc., Newark, DE, USA). Lactose concentrations were quantified by HPLC, by means of a Shimadzu HPLC system (Shimadzu Corporation, Kyoto, Japan) equipped with refractive index detector. A Hypersil APS-2 column (250 mm × 4.6 mm; ID: 5 µm; Thermo Fisher Scientific, MA, USA) was used for separation. The mobile phase was acetonitrile/water (80:20, *v*/*v*; Samchun pure chemical Co., Ltd., Pyeongtaek, Korea), with a flow rate of 1 mL/min. Column temperature was maintained at 40 °C. The amount of adsorbed lactose on the surface of ZnO NPs was calculated by subtracting unbound concentrations in the supernatants from the total lactose concentration.

### 4.6. Interactions between ZnO NPs and Mineral in Protein Matrix

The stock suspension of ZnO NPs was suspended in skim milk (1 mg/mL) or calcium (14 μg/mL of calcium in Ca(OH)_2_) solution, obtaining a final NP concentration of 0.5 mg/mL), and incubated with gentle shaking at 25 °C. After incubation for 0.02, 1, 24, 48, and 168 h, samples were centrifuged at 23,000× *g*, and washed three times with DW. The aliquots were digested, and adsorbed calcium concentrations on NP surface were determined by ICP-AES (JY2000 Ultrace; HORIBA Jobin Yvon, Longjumeau, France), as described for the ICP-AES analysis.

### 4.7. SDS-PAGE Analysis

Protein primary structural stability of protein matrices (skim milk or casein) incubated with ZnO NPs (0.5 mg/mL) was analyzed by SDS-PAGE. ZnO NPs were incubated with skim milk (1 mg/mL) or casein solution (0.35 mg/mL) by gentle shaking at 25 °C for 0.02, 1, 24, 48, and 168 h, followed by freeze-drying (Alpha 1–4 LD Plus; Martin Christ Gefriertrocknungsanlagen GmbH, Osterode am Harz, Germany).

Digestive efficiency of proteins incubated with ZnO NPs was evaluated by pepsin treatment. One milliliter of pepsin solution (32 mg/mL in DW) was added to the ZnO NPs–protein reaction solution (obtaining a final pepsin concentration of 3.2 mg/mL, equivalent to 1990 units/mL) and adjusted to a pH of 1.5 with 1 N HCl. After incubation at 37 °C for 1 h, the samples were freeze-dried. All protein samples were resuspended in DW (1 mL), and concentrations of proteins were determined by Bradford reagent (Bio-Rad, Hercules, CA, USA) prior to SDS-PAGE analysis.

Protein samples were diluted in sample buffer (4% SDS, 0.1% bromophenol blue, 10% glycerol, 0.5% β-mercaptoethanol in 50 mM Tris–HCl, pH 6.8). After heating at 95 °C for 5 min, and subsequent cooling to room temperature, samples were loaded onto 14% SDS-polyacrylamide gels.

### 4.8. CD Analysis

Conformational stability of protein matrices (skim milk or casein solution) incubated with ZnO NPs (0.5 mg/mL in protein solutions) was evaluated using CD spectroscopy (JASCO J-1500; Jasco Co., Ltd., Tokyo, Japan). A quartz cell (0.5 mm) was used for all measurements. The scanning speed was 100 nm/min, and scanning wavelengths were between 200 and 260 nm. All CD analyses were carried out at room temperature based on data from three replicate continuous scans.

### 4.9. Cytotoxicity

Human intestinal epithelial Caco-2 cells were purchased from the Korean Cell Line Bank (Seoul, Republic of Korea). The cells were cultured in MEM (Welgene, Gyeongsan, Korea), supplemented with 10% heat-inactivated FBS, 100 units/mL of penicillin, and 100 µg/mL of streptomycin (Welgene) in a humidified incubator (5% CO_2_/95% air) at 37 °C. Cells (1 × 10^4^ cells/100 μL) were cultured in 96-well plates, and treated with ZnO NPs prepared in matrices (i.e., 1 mg/mL skim milk, 0.35 mg/mL casein solution, 0.5 mg/mL lactose solution, DW, or MEM) for 24 h. Then, 10 µL of WST-1 solution (Roche, Mannheim, Germany) was added to each well. After further incubation for 4 h, absorbances at 440 nm versus 650 nm were measured using a plate reader (SpectraMax^®^ M3, Molecular Devices, Sunnyvale, CA, USA).

The released levels of LDH were measured with the CytoTox 96 Non-Radioactive Cytotoxicity assay (Promega, Madison, WI, USA). Cells (2 × 10^4^ cells/mL) were treated with ZnO NPs prepared in skim milk, casein, or lactose solution. After 24 h, the cells were detached by treatment with trypsin-ethylenediaminetetraacetic acid (EDTA). After centrifugation, aliquots (50 µL) of the supernatants were collected and placed into 96-well plates. Then, substrate solution (50 µL) was added to each well and followed by further 30 min incubation at room temperature. Finally, the absorbance at 490 nm was measured with a microplate reader (SpectraMax^®^ M3; Molecular Devices, Sunnyvale, CA, USA) after addition of a stop solution (50 μL). LDH release was expressed relative to the basal release levels from non-treated cells. In all cell experiments, protein matrices, lactose solution, DW, or MEM without NPs were used as controls.

### 4.10. Cellular Uptake

Cells (1 × 10^6^ cells/2 mL) were incubated with ZnO NPs prepared in protein matrices or lactose solution, DW or MEM for 6 h. Then, the cells were washed with phosphate-buffered saline (PBS), further treated with 5 mM EDTA for 40 s in order to detach non-specifically adsorbed NPs from the membrane surface. After washing with PBS, the cells were harvested with a scraper and prepared in de-ionized, distilled water (DDW). After centrifugation, ICP-AES (JY2000 Ultrace, HORIBA Jobin Yvon, Bensheim, Germany) analysis was performed to determine Zn concentrations, in the same manner as described for ICP-AES analysis.

### 4.11. Intestinal Transport

Human Burkitt’s lymphoma Raji B cells were purchased from the Korean Cell Line Bank. The cells were cultured in Rosewell Park Memorial Institute (RPMI) 1640 medium, containing 10% FBS, 1% non-essential amino acids, 1% L-glutamine, 100 units/mL of penicillin, and 100 μg/mL of streptomycin, in a humidified incubator (5% CO_2_/95% air) at 37 °C. An FAE model was prepared as reported by des Rieux et al. [38]. Briefly, Caco-2 cells (1 × 10^6^ cells/well) were grown on upper inserts and cultured for 14 days. Then, Raji B cells (1 × 10^6^ cells/well) in Dulbeco’s Modified Eagle’s Medium (DMEM) were added to basolateral inserts and further co-cultured for 5 days (transepithelial electrical resistance (TEER) of 150–200 Ω cm^2^). Finally, the apical medium of co-culture monolayers was replaced by different protein matrices, lactose solution, DW, or MEM, containing ZnO NPs (50 μg/mL), and the model was incubated for another 6 h.

An in vitro Caco-2 monoculture model was used to evaluate the transport of ZnO NPs by intestinal epithelium monolayer. Caco-2 cells (4.5 × 10^5^ cells/well) were seeded on upper inserts and further cultured for 21 days (TEER ≥ 300 Ω cm^2^). Then, apical medium of the monolayers was replaced with ZnO suspensions (50 μg/mL) prepared in food matrices, DW, or MEM, and incubation continued for 6 h. ICP-AES (JY2000 Ultrace, HORIBA Jobin Yvon) analysis was performed to determine Zn concentrations in basolateral solutions, in the same manner as described in “ICP-AES analysis.”

### 4.12. ICP-AES Analysis

Cell samples were pre-digested with 10 mL of ultrapure nitric acid (HNO_3_) at ~160 °C, and hydrogen peroxide (H_2_O_2_, 1 mL) solution was added and heated until the samples were colorless and clear. The digested samples were diluted with 3 mL of DDW, and total Zn concentrations were determined by ICP-AES (JY2000 Ultrace; HORIBA Jobin Yvon).

### 4.13. Statistical Analysis

Results were expressed as means ± standard deviation. One-way analysis of variance (ANOVA) with Tukey’s test were performed using SAS Ver. 9.4 statistical software (SAS Institute Inc., Cary, NC, USA) to analyze differences between control and sample groups. The threshold for statistical significance was set at *p* values of <0.05.

## 5. Conclusions

The interactions between ZnO NPs and a complex protein-containing food matrix, skim milk, were evaluated, and the interactions were compared with those with one-component matrices containing the single major components of skim milk, i.e., casein, lactose, and calcium. The interactions affected the hydrodynamic radius of ZnO NPs in both aqueous and cell culture medium in a similar manner, but positive zeta potentials of ZnO NPs shifted to negative in protein matrices and cell culture medium. ZnO NPs interacted more strongly with single-component matrices, such as casein, lactose, and calcium solutions, than with skim milk, suggesting a role of the more complex food matrix as a dispersant. However, the NP–matrix interactions did not affect cytotoxicity, cellular uptake, intestinal transport, and digestive efficiency, although modest structural deformations of proteins were induced by ZnO NPs. It may therefore be concluded that the interactions of ZnO NPs with protein matrices or other major components are not sufficiently strong to affect biological behaviors.

## Figures and Tables

**Table 1:** Hydrodynamic radii and zeta potentials of ZnO NPs suspended in distilled water (DW), Minimum Essential Medium (MEM), skim milk, lactose, casein, or calcium solutions for 1 h, followed by dilution in DW or MEM for analysis.

Matrix	Hydrodynamic Radius (nm)		Zeta Potential (mV)	
	DW	MEM	DW	MEM
DW	1957.0 ± 113.3 ^a^	2347.7 ± 347.9 ^a^	14.8 ± 0.7 ^a^	−8.4 ± 1.2 ^a^
MEM	585.6 ± 34.5 ^c^	574.0 ± 25.7 ^b^	−24.2 ± 1.4 ^d^	−9.2 ± 1.0 ^a^
Skim milk	1123.0 ± 266.1 ^ac^	1176.7 ± 120.8 ^ab^	−18.0 ± 0.1 ^c^	−9.5 ± 1.4 ^a^
Casein	2417.0 ± 744.1 ^b^	2667.0 ± 1094.1 ^a^	−15.1 ± 0.3 ^c^	−10.0 ± 0.5 ^a^
Lactose	1509.0 ± 462.1 ^a^	1800.3 ± 573.5 ^ab^	15.6 ± 0.3 ^a^	−9.6 ± 0.5 ^a^
Calcium	3186.4 ± 319.6 ^d^	2301.8 ± 477.5 ^a^	9.3 ± 3.4 ^b^	−9.5 ± 2.6 ^a^

Significant differences between differently dispersed matrices are indicated by lower-case letters (a–d) in the same column (*p* < 0.05).
